# ARID1B基因缺失促进NSCLC细胞的增殖、迁移和侵袭能力的研究

**DOI:** 10.3779/j.issn.1009-3419.2025.101.04

**Published:** 2025-03-20

**Authors:** Linlin ZHU, Xuchao ZHANG

**Affiliations:** ^1^510080 广州，南方医科大学附属广东省人民医院（广东省医学科学院）（朱琳琳，张绪超）; ^1^Guangdong Provincial People's Hospital (Guangdong Academy of Medical Sciences), Southern Medical University, Guangzhou 510080, China; ^2^510515 广州，南方医科大学第二临床医学院（朱琳琳，张绪超）; ^2^The Second School of Clinical Medicine, Southern Medical University, Guangzhou 510515, China; ^3^510080 广州，南方医科大学附属广东省人民医院（广东省医学科学院），医学研究部，广东省肺癌转化医学重点实验室（朱琳琳，张绪超）; ^3^Guangdong Provincial Key Laboratory of Translational Medicine in Lung Cancer, Medical Research Center, Guangdong Provincial People's Hospital (Guangdong Academy of Medical Sciences), Southern Medical University, Guangzhou 510080, China

**Keywords:** ARID1B, 肺肿瘤, 增殖, 迁移, MAPK, 转录组学分析, ARID1B, Lung neoplasms, Proliferation, Migration, MAPK, RNA-seq

## Abstract

**背景与目的:**

SWI/SNF染色质重塑复合物（switch/sucrose nonfermentable chromatin-remodeling complex）的异常与多种癌症密切相关，ARID1B（AT-rich interaction domain 1B）是SWI/SNF复合物的核心亚基之一。ARID1B基因突变或拷贝数缺失与DNA损伤反应受损、染色质可及性改变有关。然而，ARID1B缺失是否影响非小细胞肺癌（non-small cell lung cancer, NSCLC）细胞的增殖、迁移和侵袭能力及其分子机制仍缺乏系统研究。本研究旨在揭示ARID1B基因缺失对NSCLC细胞恶性表型的调控作用及其分子机制。

**方法:**

通过公共数据库分析ARID1B基因与肺癌患者预后之间的相关性以及其在肺癌组织中的表达水平。CRISPR/Cas9（clustered regularly interspaced short palindromic repeat）技术构建ARID1B基因稳定敲除（knockout, KO）的细胞株。采用平板克隆实验检测细胞增殖情况，Transwell细胞迁移、侵袭实验检测细胞迁移能力变化。RNA-Seq进行差异基因的表达、富集分析。利用蛋白印迹（Western blot, WB）验证ARID1B基因敲除效果，检测上皮间充质转化（epithelial-mesenchymal transition, EMT）标志物、促分裂素原活化蛋白激酶（mitogen-activated protein kinases, MAPK）信号通路相关蛋白变化。构建裸鼠成瘤模型，并比较对照和ARID1B缺失细胞的成瘤能力。

**结果:**

低ARID1B表达与肺癌患者不良预后有显著关联，其总生存期较短。ARID1B在肺癌细胞中的表达水平较正常细胞显著降低。ARID1B缺失型细胞增殖、迁移和侵袭能力增强。动物实验中，ARID1B基因缺失组成瘤速度加快。RNA-Seq结果进行富集分析可见差异基因主要在MAPK、磷脂酰肌醇3-激酶/蛋白激酶B（phosphoinositide 3-kinase/protein kinase B, PI3K/Akt）等信号通路富集。WB证明ARID1B基因缺失细胞E-cadherin、N-cadherin、Vimentin表达发生变化，MAPK、p-MAPK表达增加。

**结论:**

成功建立A549-ARID1B KO和PC9-ARID1B KO细胞株，ARID1B缺失细胞株在体外、体内生物学行为水平和转录组测序水平均提示具有高迁移、侵袭、增殖潜能。EMT标志物表达的变化、MAPK信号通路的激活提示ARID1B缺失型NSCLC可能的转移机制。

## Abstract (en)

**Background and objective:**

Abnormalities of the switch/sucrose nonfermentable (SWI/SNF) chromatin-remodeling complex are closely related to various cancers, and ARID1B (AT-rich interaction domain 1B) is one of the core subunits of the SWI/SNF complex. Mutations or copy number deletions of the ARID1B gene are associated with impaired DNA damage response and altered chromatin accessibility. However, whether ARID1B deficiency affects the proliferation, migration and invasion abilities of non-small cell lung cancer (NSCLC) cells and its molecular mechanisms remain poorly understood. This study aims to reveal the regulatory role of ARID1B gene deletion on the malignant phenotype of NSCLC cells and its molecular mechanism.

**Methods:**

Online databases were used to analyze the relationship between ARID1B and the prognosis of patients with lung cancer, and the expression levels of ARID1B in lung cancer tissues. The CRISPR/Cas9 (clustered regularly interspaced short palindromic repeat) technology was employed to construct stable ARID1B gene knockout (KO) cell lines. The plate colony formation assay was used to detect cell proliferation, and the Transwell cell migration and invasion assays were used to detect changes in cell migration ability. RNA-Seq was utilized for the expression and enrichment analysis of differentially expressed genes. Western blot (WB) was used to verify the knockout effect of the ARID1B gene and to detect the expression changes of epithelial-mesenchymal transition (EMT) markers and mitogen-activated protein kinases (MAPK) signaling pathway-related proteins. Nude mouse tumor models were constructed and the tumorigenic abilities of control and ARID1B-deficient cells were compared.

**Results:**

Patients with low ARID1B expression have poor overall survival. ARID1B is differentially expressed in lung cancer and normal tissues, and its expression level being lower in cancer cells. ARID1B-deficient cells had significantly enhanced in vitro proliferation, migration and invasion abilities. In animal experiments, the tumor formation speed of ARID1B gene deficient cells was significantly accelerated. Enrichment analysis of RNA-Seq results revealed that the differentially expressed genes were mainly enriched in MAPK, phosphoinositide 3-kinase-protein kinase B (PI3K/Akt) and other signaling pathways. WB experiments demonstrated that the expressions of E-cadherin, N-cadherin and Vimentin changed in ARID1B gene deficient cells, and the expressions of MAPK and p-MAPK was increased.

**Conclusion:**

The A549-ARID1B KO and PC9-ARID1B KO cell lines were successfully established. The ARID1B-deficient cell lines demonstrated high migration, invasion and proliferation potential at both in vitro and in vivo biological behavior levels and at the transcriptome sequencing level. The changes in the expression of EMT markers and the activation of the MAPK signaling pathway suggest possible metastasis mechanisms of ARID1B-deficient NSCLC.

GLOBOCAN 2024全球癌症统计数据^[1]^显示，肺癌持续位居全球癌症发病谱及死亡谱首位，2024年全球新发肺癌病例达2,480,675例，占恶性肿瘤总发病数的12.4%。其中非小细胞肺癌（non-small cell lung cancer, NSCLC）作为最主要的组织学亚型，约占肺癌病例的85%，其病理进程复杂且预后不良。尽管近年来靶向治疗和免疫治疗在NSCLC临床管理中取得突破性进展，但由于肿瘤异质性和治疗耐药性问题，患者的5年生存率仍然不容乐观。因此，系统地解析NSCLC的分子病理机制并识别新型治疗靶标，已成为改善临床预后的关键科学问题。

表观遗传调控异常在恶性肿瘤发生发展中具有核心作用，其中SWI/SNF染色质重塑复合物通过动态调控核小体空间构象，在DNA损伤修复、细胞周期调控及上皮间充质转化（epithelial-mesenchymal transition, EMT）等关键生物学过程中发挥重要功能^[2]^。值得注意的是，该复合物亚基在恶性肿瘤中普遍存在高度异质性突变^[3]^，其突变频率与磷脂酰肌醇3-激酶/蛋白激酶B/哺乳动物雷帕霉素靶蛋白（phosphoinositide 3-kinase/protein kinase B/mammalian target of rapamycin, PI3K/Akt/mTOR）通路关键节点基因及TP53等经典抑癌基因相当^[4]^。作为SWI/SNF复合物的核心组成亚基，ARID1B通过介导染色质拓扑重构调控特定基因簇的转录活性^[5,6]^。目前多项研究^[7⇓-9]^已证实ARID1B在神经母细胞瘤、结直肠癌及乳腺癌等实体瘤中呈现双重调控作用：其表达缺失既可诱导基因组不稳定性，又能通过重塑肿瘤微环境促进恶性表型进展。有研究^[6]^表明，在NSCLC模型中，ARID1B缺陷不仅导致DNA损伤应答机制受损，还可通过激活固有免疫信号通路影响肿瘤免疫原性。然而，ARID1B在NSCLC增殖侵袭等恶性生物学行为中的调控机制及其与表观遗传网络互作的关系尚未阐明。

本研究通过整合分子生物学实验及转录组学数据，系统探究ARID1B基因缺失对NSCLC细胞增殖、迁移、侵袭能力的影响，并进一步揭示其潜在的分子机制，研究结果将为NSCLC的治疗提供新的思路和靶点。

## 1 材料与方法

### 1.1 在线数据库分析

使用Kaplan-Meier在线数据库（ http://kmplot.com/），分析肺癌中ARID1B表达对预后的影响，阈值P<0.05。使用GEPIA在线分析工具（ http://gepia.cancer-pku.cn/）分析ARID1B在肺腺癌和正常组织中的表达量数据，阈值P<0.01。使用The Human Protein Atlas（ https://www.proteinatlas.org/）提供的ARID1B在肺腺癌和正常组织中表达的免疫组化（immunohistochemistry, IHC）染色图像显示ARID1B蛋白在肺腺癌和正常组织的表达定位和强度。

### 1.2 细胞培养

本实验使用的肺腺癌细胞系NCI-A549、NCI-PC9来自于广东省肺癌研究所（广东省肺癌转化医学重点实验室），所有实验用的细胞系经STR鉴定正确、支原体检测没有污染后，使用含10%胎牛血清（Gibco, 1932594C）的RPMI-1640培养基（Gibco, C22400500BT）进行细胞培养，细胞生长条件为5% CO₂、37 ^o^C的细胞培养箱。

### 1.3 Cas9靶位点的选择

靶位点选择在基因编码序列（coding sequence, CDS）的前2/3区域并且在起始密码子之后，破坏重要的结构域和/或所有的可变剪接形式。靶点也可选择在内含子和外显子交界处，以破坏基因的剪接。原则上不要选择5’-UTR（Untranslated Region）和3’-UTR。可参考如下的Cas9靶位点预测网站： http://zifit.partners.org/ZiFiT/CSquare9Nuclease.aspx。

### 1.4 sgRNA质粒载体构建

先磷酸化和退火每对寡核苷酸链（NEB, M0201S）；再使用内切酶BsmBI-v2（NEB, R0739S）对质粒空载lentiCRISPR v2-Blast（Addgene, 83480）进行限制性核酸内切酶反应，利用凝胶提取试剂盒（Omega, D2500-02）对酶切的空载质粒进行凝胶纯化，将退火寡聚物以1:200稀释到无菌水或EB缓冲液中，使用T4 DNA连接酶（NEB, M0202V）将纯化的质粒空载与退火寡核苷酸链Oligo连接，质粒转化至Stbl3感受态细胞（Genesand, XK002-01），转化后的感受态细胞保种、根据实验需要提取质粒。sgRNA序列如表1所示，基因组DNA扩增引物序列如表2所示。

**表1:** sgRNA序列

Genes		Sequence (5’-3’)
ARID1B-sg1	Top (5’-3’) Bottom ( 5’-3’)	TGAGTGCAAGATCGAACGTG CACGTTCGATCTTGCACTCA
ARID1B-sg2	Top (5’-3’) Bottom (5’-3’)	TCCCGGAGTTTAATAATTAC GTAATTATTAAACTCCGGGA
ARID1B-sg3	Top (5’-3’) Bottom (5’-3’)	CAGCAGAGCAGTCCGTACCC GGGTACGGACTGCTCTGCTG

sgRNA: single-guide RNA

**表2:** 基因组DNA扩增引物序列

Genes		Sequence (5’-3’)
ARID1B-sg1	Forward (5’-3’) Reverse (5’-3’)	CCAATGATCCTGCCGTGTTTTTCACTAGG ATCCCTTAACACTGCCAGTGGGCGTCCAA
ARID1B-sg2	Forward (5’-3’) Reverse (5’-3’)	AACATGGAGGCGCCAAGGACAGTGCT ATCATGGGGCTGGGCGAGGTGAGCAGCTGATTGA
ARID1B-sg3	Forward (5’-3’) Reverse (5’-3’)	CTTTAATGTCTTGCTGGATGTTTTGTCAGG TTCTGTCCACTTTAAGAAGCTCACCTTTCC

### 1.5 慢病毒包装

配制Mix1：目的质粒+psPAX2（包装质粒）+pMD2.G（包膜质粒）+opti-MEM+P3000TM增强剂；Mix2：Lipo3000+opti-MEM，上面两个Mix分别孵育5 min。将两个Mix混合并孵育10-15 min。Mix混合物加入到6孔板opti-MEM细胞培养基中。转染48 h后，收获含病毒的上清于50 mL离心管中，病毒液放入-80 °C冰箱保存。

### 1.6 ARID1B基因稳定敲除的细胞株构建

慢病毒感染目的细胞， 杀稻瘟菌 素（HANBIO, HB-BSD）筛选，病毒感染细胞后1周进行抗生素筛选。加药约48 h实验组细胞有部分存活，则病毒感染成功，将经过 杀稻瘟菌 素筛选的细胞扩增培养。

筛选携带靶位点突变的单个克隆细胞，有限稀释法筛选单个克隆细胞，送Sanger测序进行单克隆株基因型鉴定，对鉴定为成功敲除的阳性克隆，收细胞沉淀进行蛋白印迹（Western blot, WB）验证。WB验证成功鉴定为目的蛋白完全缺失的阳性克隆继续传代扩增。

### 1.7 WB检测蛋白表达水平

使用RIPA裂解液（Beyotime, P0013B）提取细胞沉淀总蛋白，BCA试剂盒（Solarbio, PC0020）检测蛋白浓度。10% SDS-PAGE凝胶（Vazyme, E303-01）电泳，分离蛋白样品，电泳后，恒流将蛋白转至PVDF膜（0.20 μm, Merck millipore, ISEQ00010），TBST洗膜，脱脂奶粉封闭1 h，4°C过夜孵育一抗ARID1B（1:1000, CST, 92964）、E-cadherin（1:1000, CST, 3195）、N-cadherin（1:1000, CST, 13116）、Vimentin（1:1000, CST, 5741）、MAPK（1:1000, CST, 4696）、phospho-p44/42 MAPK（1:1000, CST, 4376），室温孵育二抗anti-rabbit IgG（1:2000, CST, 7074）2 h，化学发光底物（enhanced chemiluminescence, ECL）（NCM, P10300）显影液显影。

### 1.8 细胞克隆形成实验

实验所需细胞株在T25细胞培养瓶培养，使用Trypsin胰酶消化细胞，细胞消化完成后在显微镜下进行细胞计数，最终结果使用的是每孔1×10^3^个细胞的密度接种于6孔板。在标准培养条件下进行常规培养，并定期更换培养液，同时密切观察细胞生长状态。培养过程持续10 d左右，或者至大多数单个克隆中的细胞数量约50个。丢弃细胞完全培养基，用PBS洗涤细胞，4%多聚甲醛（Beyotime, P0099）固定30 min，用PBS洗涤，加入1 mL结晶紫染色液（Beyotime, C0121）染色，时间为15 min。染色结束后，用PBS反复洗涤至干净，以去除未结合的染色液，随后晾干。在Image J中计算细胞克隆形成数，并根据公式克隆形成率=（克隆形成数/接种细胞数）×100%，计算克隆形成率。

### 1.9 Transwell细胞迁移/侵袭实验

细胞消化、用无血清培养基重悬计数，下室加入700 µL含10%胎牛血清的完全培养基；小室内加入400 μL含8.0×10^4^个细胞的细胞悬液，常规培养24 h；多聚甲醛（Biosharp, BL539A）固定细胞，PBS清洗两次，结晶紫（Beyotime/碧云天，C0121-500 mL）染色15 min（小室内和孔内都加染液），去除染液，PBS清洗两次。轻轻擦去上室面未迁移的细胞。显微镜下观察、拍照、计数。细胞侵袭实验其余步骤同迁移实验，除第一步需用50 μL Matrigel 1:8稀释液包被Transwell小室底部膜的上室面，置入小室的孔板放入37 °C培养箱12 h，吸出小室中还没有干的残余的液体，进行水化时每孔可以加入70 μL含10%血清的1640完全培养基，37 °C、30 min，水化基底膜。小室内加入400 μL含8.0×10^4^个细胞的细胞悬液，常规培养48 h。

### 1.10 体内成瘤实验

本研究严格遵循国家关于动物管理和使用的法规及标准，并符合实验动物伦理学委员会制定的伦理要求（伦理批号：No.GDREC2019515H）。实验遵循美国国立卫生研究院（National Institutes of Health, NIH）《实验动物护理和使用指南》，经广东省医学实验动物中心动物护理和使用指南委员会批准。5-6周龄同性别裸鼠（广东省医学实验动物中心）随机分组进行皮下成瘤，每组5只，至少进行3次重复实验。使用1.25%阿佛丁（易核，M2910）经腹腔注射进行麻醉，取2×10^6^个A549-ARID1B KO、PC9-ARID1B KO细胞和亲本对照组细胞，分别皮下注射到裸鼠左部皮肤处，每组5只。监测小鼠活动、饮食和体重，2周后收获瘤体，使用球后静脉空气注射行安乐死。观察肿瘤块大小，肿瘤块在4%多聚甲醛固定液中固定24 h，冲洗30 min，保存在75%酒精中。瘤体体积（V）=长×宽^2^/2。

### 1.11 转录组测序及生物信息学分析

#### 1.11.1 转录组测序

使用Trizol（Thermo Fisher, 15596026）提取A549-ARID1B KO细胞、PC9-ARID1B KO细胞、A549空载对照细胞、PC9空载对照细胞总RNA，提取过程中保证RNA质量，样品检测合格之后，进行样品的文库构建。本研究采用M-MuLV逆转录酶体系合成cDNA第一条链；利用dNTPs作为底物合成cDNA第二条链；对双链cDNA进行纯化，随后依次进行末端修复、加A尾和连接测序接头；筛选出长度在200 bp左右的cDNA，对其进行聚合酶链式反应（polymerase chain reaction, PCR）扩增，纯化PCR产物，最终构建测序文库。使用Illumina PE150进行测序。

#### 1.11.2 差异表达基因获取及富集分析

本研究的基因差异表达分析所使用的数据，来源于基因表达水平分析所获得的Reads Count数据。运用DESeq2算法对样本实施差异基因检测，借助R包在|log2(foldchange)|>1且P<0.05的条件下，确定差异表达的基因。在完成差异基因的筛选工作之后，将依据其检测结果，进一步开展基因本体论（Gene Ontology, GO）富集分析。与此同时，针对这些差异基因，还将进行京都基因与基因组百科全书（Kyoto Encyclopedia of Genes and Genomes, KEGG）富集分析。

### 1.12 统计学分析

计量数据以（Mean±SD）表示，每组数据至少重复3次，计量资料两组间比较采用t检验，使用IBM SPSS 22.0、GraphPad Prism 8.2统计分析软件进行统计学处理。P<0.05表示差异具有统计学意义，所有检验均为双侧检验。

## 2 结果

### 2.1 在线数据库分析ARID1B表达与肺癌预后的关系以及ARID1B在肺腺癌组织中的表达量分析

使用Kaplan-Meier在线数据库检查ARID1B过表达与低表达与肺癌预后之间的关系。在肺癌患者中（n=1411），低ARID1B表达水平显示出显著不良的总生存期（overall survival, OS）[风险比（hazard ratio, HR）=0.85，95%置信区间（confidence interval, CI）=0.73-0.99]（图1A）。使用GEPIA在线数据库分析ARID1B在肺癌组织中的表达量数据，箱形图显示ARID1B在肺腺癌组织和正常组织中存在差异表达，且在肿瘤组织中表达水平较低（图1B）。The Human Protein Atlas数据库的免疫组化染色图像显示ARID1B在肺腺癌组织和正常组织中存在差异表达，在肺癌细胞中表达水平更低（图1C）。

**图1:**
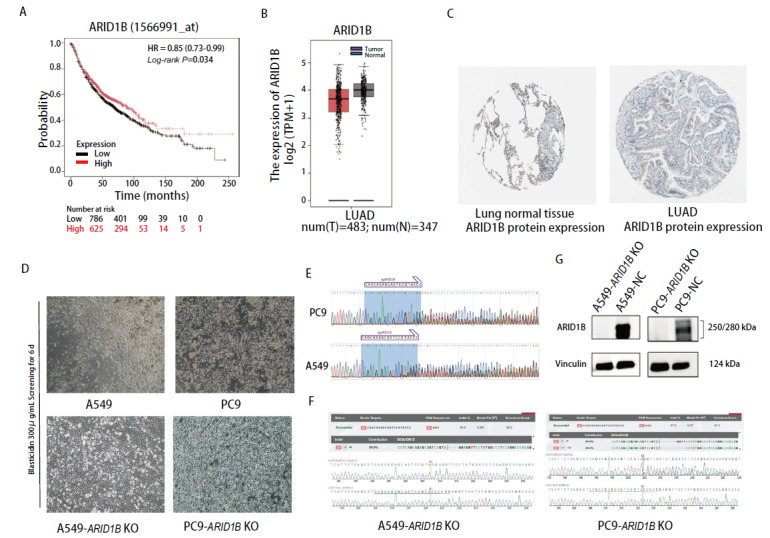
ARID1B基因表达水平对肺癌患者OS的影响、ARID1B在肺癌和正常组织中的表达水平比较以及肺癌细胞株A549-ARID1B KO、PC9-ARID1B KO细胞模型的建立。A：使用Kaplan-Meier在线数据库检查ARID1B表达量与肺癌预后之间的关系；B：使用GEPIA在线分析工具分析ARID1B在肺腺癌组织和正常组织中的表达量数据；C：The Human Protein Atlas提供的ARID1B在肺腺癌组织和正常组织中表达的IHC染色图像；D：未感染病毒的对照组和感染过病毒的实验组，加入等量浓度的 杀稻瘟菌 素筛选；E：通过 杀稻瘟菌 素抗性筛选获得多克隆阳性细胞池Sanger测序结果；F：进一步的单克隆敲除株分选，经Sanger测序鉴定敲除类型；G：WB验证ARID1B蛋白水平的缺失。

### 2.2 ARID1B基因敲除细胞株的构建

将编码Cas9蛋白和sgRNA的质粒用Lipo3000包装成慢病毒， Cas9 蛋白在sgRNA引导下对 细胞基因组的靶标基因进行切割。利用 慢病毒感染A549、PC9细胞，目的细胞携带抗生素筛选抗性（图1D）。通过抗性筛选获得多克隆阳性细胞池（图1E），通过有限稀释单克隆挑选方式进行进一步的单克隆敲除株分选，再逐一鉴定敲除类型（图1F），得到基因组水平发生移码突变的单克隆细胞株，扩增培养，用WB验证蛋白水平的缺失，最终得到ARID1B蛋白完全敲除的细胞（图1G）进行后续实验。以上结果表明成功建立ARID1B蛋白缺失的A549-ARID1B KO、PC9-ARID1B KO细胞株。

### 2.3 ARID1B基因敲除促进NSCLC细胞体外增殖

为了探索ARID1B缺失对NSCLC增殖能力的影响，利用A549和PC9细胞构建ARID1B敲除细胞株，利用平板克隆形成实验检测细胞的增殖能力。A549-ARID1B KO组的克隆形成率为（15.39%±2.11%）、PC9-ARID1B KO组为（17.28%±1.27%），明显多于相应对照组[A549-NC (Negative Control): (2.89%±0.70%); PC9-NC: (7.27%±1.04%), P<0.05]。上述结果提示ARID1B缺失细胞组的细胞增殖能力比相应对照组的增殖能力明显增加（图2）。因此，ARID1B缺失促进NSCLC细胞A549和PC9的增殖。

**图2:**
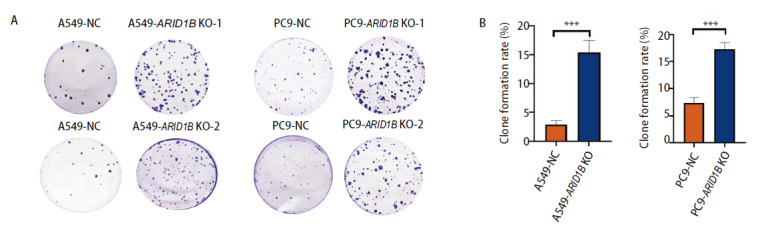
NSCLC细胞中ARID1B基因敲除促进细胞体外增殖。A：克隆形成实验检测A549-ARID1B KO、A549-NC、PC9-ARID1B KO、PC9-NC细胞的克隆形成能力；B：克隆形成实验的克隆形成率统计图。***P<0.001。

### 2.4 ARID1B基因敲除促进NSCLC细胞体外迁移、侵袭

为了探索ARID1B缺失对NSCLC迁移能力的影响，我们利用A549-ARID1B KO和PC9-ARID1B KO细胞株进行了Transwell迁移实验、侵袭实验。Transwell迁移实验可见A549-ARID1B KO组、PC9-ARID1B KO组的迁移细胞数分别为（1738.00±95.26）和（803.30±33.23），明显多于相应对照组[A549-NC: (665.30±138.30); PC9-NC: (296.80±61.32), P<0.05]。结果说明ARID1B基因的缺失可以显著促进A549和PC9细胞的迁移（图3A）。同时，我们利用A549-ARID1B KO细胞株以及PC9-ARID1B KO细胞株分别进行了Transwell侵袭实验，并在48 h后比较两组细胞与对照组之间的细胞侵袭能力是否具有差异。结果提示A549-ARID1B KO组、PC9-ARID1B KO组的侵袭细胞数分别为（1209.00±86.70）和（1381.00±113.60），明显多于相应对照组[A549-NC: (447.70±84.84); PC9-NC: (307.30±51.48), P<0.05]，可见ARID1B基因缺失组与对照组相比，细胞侵袭速度明显增加（图3B），说明ARID1B缺失显著促进NSCLC细胞的体外迁移、侵袭能力。

**图3:**
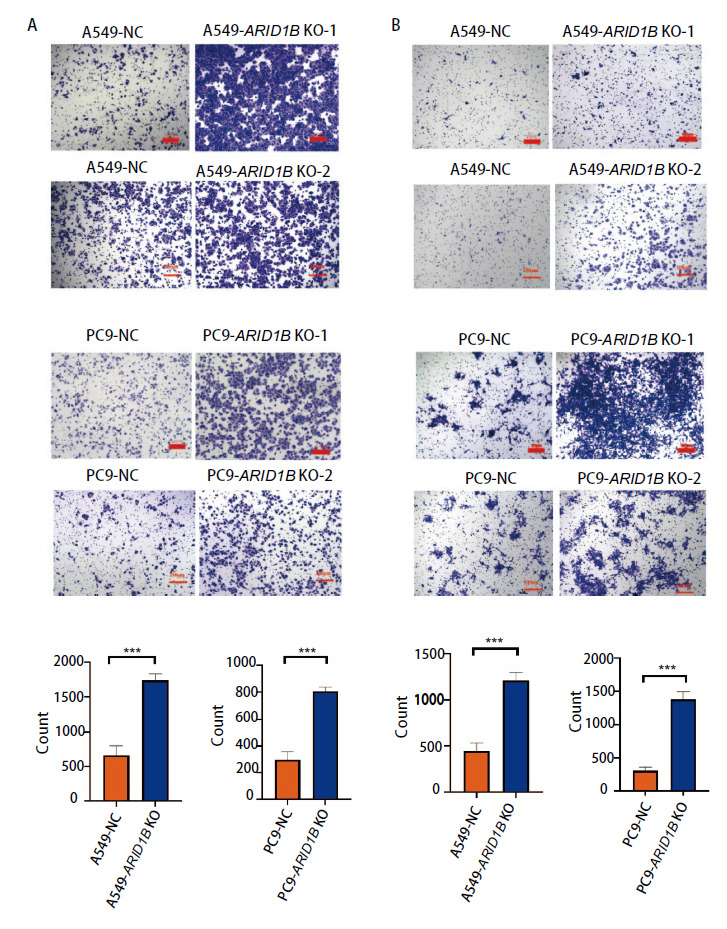
NSCLC细胞中ARID1B基因敲除促进细胞体外迁移、侵袭。A：Transwell实验表明ARID1B缺失突变细胞组的迁移能力强于空载对照组；B：Transwell实验表明ARID1B缺失突变细胞组的侵袭能力强于空载对照组。柱状图展示了Transwell迁移和侵袭后细胞数目统计数据。***P<0.001。

### 2.5 ARID1B表达缺失促进NSCLC细胞体内增殖

利用A549-ARID1B KO和PC9-ARID1B KO细胞株进行体内成瘤实验（图4），结果发现ARID1B缺失细胞组成瘤体积[A549-ARID1B KO: (410.03±172.26) mm^3^; PC9-ARID1B KO: (188.64±85.95) mm^3^]明显大于对照组[A549-NC: (58.84±30.47) mm^3^; PC9-NC: (89.60±33.98) mm^3^]（P<0.05）。ARID1B缺失细胞组瘤体重量[A549-ARID1B KO: (0.42±0.11) g; PC9-ARID1B KO: (0.37±0.08) g]明显大于对照组[A549-NC: (0.07±0.01) g; PC9-NC: (0.08±0.02) g]（P<0.05），因此，ARID1B缺失促进A549、PC9细胞体内增殖。

**图4:**
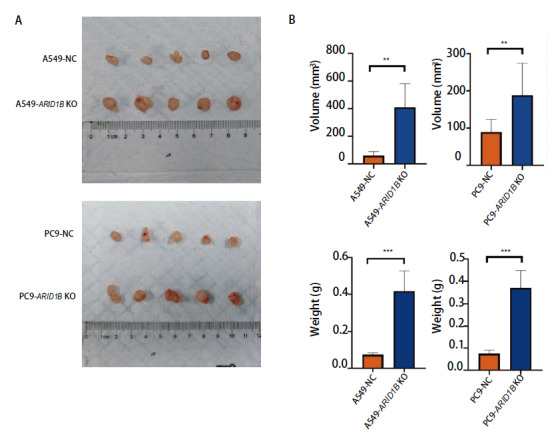
敲除A549、PC9细胞中的ARID1B基因促进其在动物体内的增长。A：种植肿瘤细胞14 d后，从裸鼠皮下取出的肿瘤；B：14 d肿瘤体积、重量统计图。与对照组比较，基因敲除组瘤体体积、重量明显增加。**P<0.01；***P<0.001。

### 2.6 A549空载对照细胞与A549-ARID1B

KO、PC9空载对照细胞与PC9-ARID1B KO细胞的转录组学测序分析

#### 2.6.1 差异基因筛选

为了对比A549-ARID1B KO细胞和PC9-ARID1B KO细胞与空载对照细胞在基因表达层面的差异，采用转录组测序技术进行分析。

采用DESeq2软件包对差异表达基因进行筛选，设定筛选条件为P<0.05且|log_2_(foldchange)|>1。分析结果显示，与空载对照细胞相比，A549-ARID1B KO细胞中共鉴定出3731个差异表达基因，其中1389个基因呈现上调表达，2342个基因则表现为下调表达。进一步的聚类分析表明，两组重复样品在差异基因表达模式上具有高度一致性。在PC9-ARID1B KO细胞中，相对于空载对照细胞，共检测到2497个差异表达基因，包括1553个上调基因和944个下调基因（图5A、5B）。将A549-ARID1B KO、PC9-ARID1B KO与空载对照细胞比较的两组差异基因取交集后得到599个交集基因。共同上调的基因190个和共同下调的基因241个（图5C）。差异表达基因聚类分析显示ARID1B基因缺失细胞和空载对照细胞间的基因表达谱存在显著差异（图5D）。

**图5:**
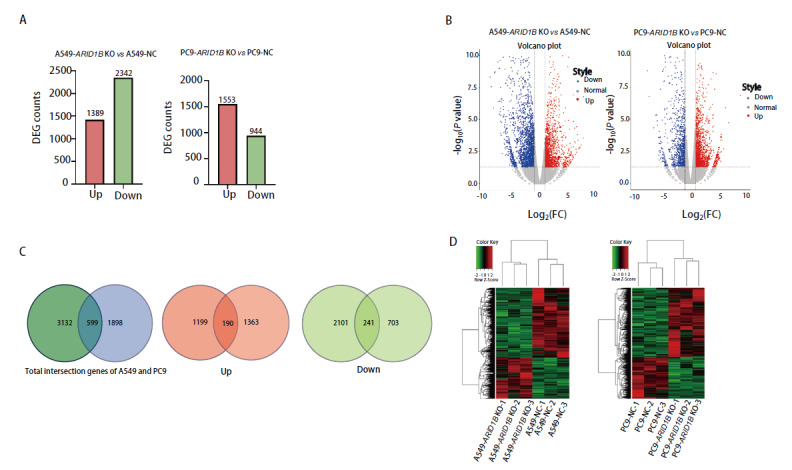
RNA-seq分析差异基因表达情况。A：两组细胞差异基因柱状统计图；B：两组细胞差异基因火山图；C：两组差异基因取交集后得到599个交集基因。共同上调的基因190个和共同下调的基因241个；D：两组细胞差异基因聚类热图。

#### 2.6.2 GO富集分析、KEGG富集分析

经GO富集分析，与空载对照细胞相比，A549-ARID1B KO、PC9-ARID1B KO细胞的差异表达基因在细胞组分上主要分布于细胞外基质、细胞基部等区域；在分子功能方面，主要集中在离子通道活性、GTP结合等；在生物过程上，主要涉及细胞黏附、细胞生长、白细胞迁移等（图6A）。

**图6:**
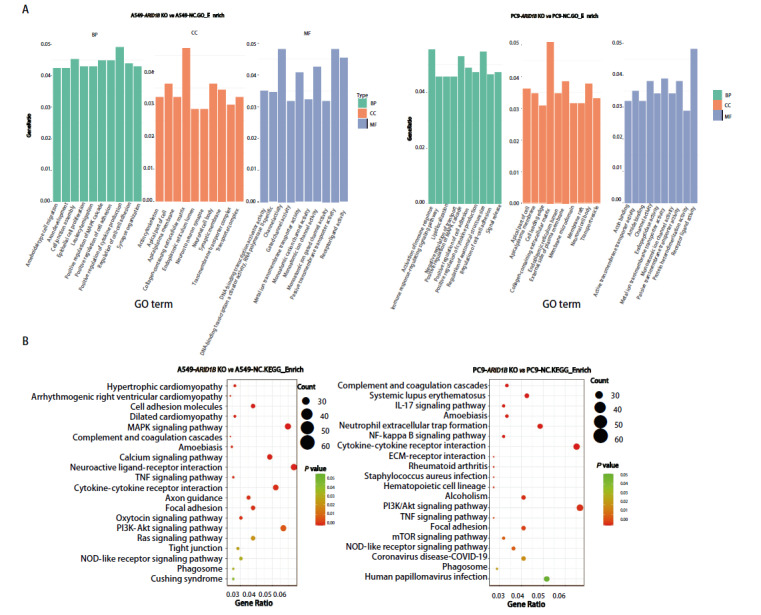
对两组细胞的差异表达基因开展GO富集分析及KEGG通路富集分析。A：GO富集分析；B：KEGG通路富集分析。

为深入探究ARID1B基因缺失细胞与对照细胞在基因表达层面的差异，本研究对两组细胞的差异表达基因进行了KEGG通路富集分析。分析结果显示，这些差异基因在多个关键信号通路中呈现出显著的富集现象。主要包括神经活性配体-受体互作、MAPK信号通路、PI3K/Akt信号通路、细胞因子-细胞因子受体互作、钙信号通路、细胞黏附分子、黏着斑以及轴突导向等（图6B）。

### 2.7 ARID1B通过激活MAPK信号通路调节细胞增殖和迁移

为了进一步探讨ARID1B促进NSCLC增殖的机制，前期我们利用RNA-seq对下游通路进行了分析，发现ARID1B可能调节MAPK信号通路。因此，我们利用WB检测A549-ARID1B KO、PC9-ARID1B KO细胞和空载对照细胞MAPK信号通路相关蛋白表达。结果提示，与对照组相比，ARID1B表达缺失后，MAPK、p-MAPK表达明显增加（P<0.05）（图7A），说明ARID1B可能通过激活MAPK通路调节NSCLC细胞的增殖和迁移。同时，我们推测NSCLC细胞通过EMT，获得了迁移与侵袭等间质表型。我们检测了ARID1B缺失对NSCLC细胞EMT过程的影响。WB的结果显示ARID1B蛋白缺失下调E-cadherin的表达，上调N-cadherin和Vimentin的表达（图7B）。

**图7:**
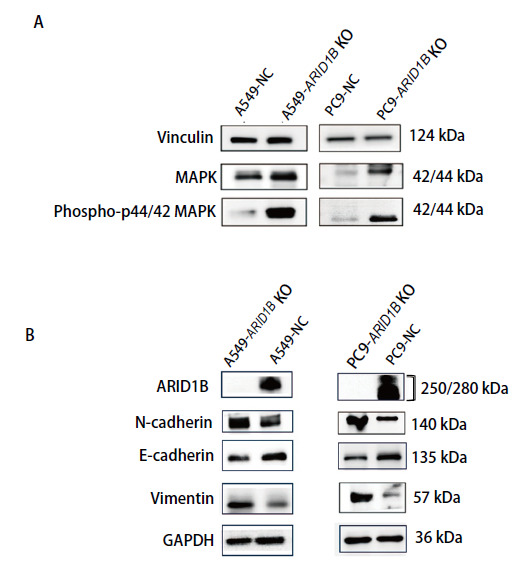
蛋白印迹实验。A：ARID1B缺失细胞MAPK信号通路相关蛋白的表达变化；B：ARID1B对NSCLC细胞上皮间充质转化标志物表达的影响。

## 3 讨论

近年来，SWI/SNF复合体各亚单位的突变被证实与多种恶性肿瘤的发生、发展及治疗耐药密切相关，它通过操纵染色质组织允许某些基因在不同组织类型中的选择性表达^[10]^。ARID1A和ARID1B是SWI/SNF复合体必需的DNA结合亚基^[11]^。核心SWI/SNF复合体亚基表达异常的去分化和未分化子宫内膜癌和卵巢癌显示大约一半的ARID1B表达缺失，而另一半SMARCA4表达缺失^[12,13]^。这种分布也反映在卵巢DDC/UDC中，其中约一半显示ARID1B缺失^[14]^。Helming等和DRIVE项目^[15]^表明，ARID1B是ARID1A密切相关的旁系同源物，是ARID1A突变细胞中的合成致死靶标。由于这些是cBAF复合物中互斥且部分冗余的亚基，因此认为细胞需要任一组分才能存活。两者的敲低都会导致染色质可及性降低，尤其是在增强子周围，目前尚不清楚这如何转化为细胞表型^[7]^。本研究系统揭示了ARID1B基因缺失在NSCLC恶性表型调控中的关键作用。Kaplan-Meier生存分析显示，ARID1B低表达的NSCLC患者OS显著缩短，这一发现与既往研究^[16,17]^报道的SWI/SNF复合物成员（如ARID1A、SMARCA4）在肿瘤中的抑癌功能具有一致性。

CRISPR-Cas9系统最初是在细菌中发现的一种免疫机制，用于抵御外来的病毒和质粒。CRISPR-Cas9基因编辑技术能够快速、高效地定位并编辑目标基因。利用此方法，成功获得ARID1B基因完全缺失的A549、PC9细胞。我们的体外实验结果显示，SWI/SNF复合体突变尤其是ARID1B亚基失活突变的细胞在体外的增殖能力、迁移和侵袭能力强于复合体野生型细胞。

本研究旨在探索A549-ARID1B KO和PC9-ARID1B KO细胞株的基因表达谱改变，通过转录组测序分析ARID1B基因缺失细胞株与空载对照细胞。在A549-ARID1B KO细胞中，共鉴定出3731个差异表达基因，其中1389个上调，2342个下调。而在PC9-ARID1B KO细胞中，共检测到2497个差异表达基因，1553个上调， 944 个下调。

经GO分析，差异基因参与的生物过程集中在细胞黏附、生长及白细胞游走等方面，这些过程对肿瘤细胞的远处转移及定植具有重要意义。在KEGG富集分析中，差异基因主要集中在神经活性配体-受体相互作用、MAPK信号通路、细胞黏附分子等信号通路。

MAPK信号通路是真核生物信号传递网络中的重要途径之一。MAPK家族中研究最为广泛的是ERK1/ERK2激酶，激活的ERK1/2能够继续磷酸化ELK1、ETS、FOS、JUN、MYC和SP1等转录因子，诱导与细胞周期、细胞增殖有关的基因表达。例如，TC2N基因通过激活MAPK/ERK通路促进宫颈癌细胞增殖，敲减TC2N基因可显著抑制该通路活性。此外，MAPK/ERK信号通路的激活能够抵消SFRP1过表达对结直肠癌细胞迁移与侵袭的抑制效应，这一现象揭示了该信号通路对结直肠癌细胞的增殖及侵袭具有促进作用^[18]^。本研究转录组测序分析提示MAPK信号通路中Ras/Raf/MEK/ERK途径关键分子p21激活激酶 3（p21 activated kinase 3, PAK3）显著升高，进一步支持了ARID1B基因缺失促进NSCLC转移的机制。

此外，敲除ARID1B显著促进了NSCLC细胞的增殖、迁移、侵袭的EMT表型。在本研究中，通过敲除NSCLC细胞内的ARID1B蛋白表达，利用WB技术检测MAPK信号通路相关蛋白的表达变化，初步揭示ARID1B对肺癌细胞生物学行为的影响可能与MAPK信号通路的激活有关。同时，EMT标志物（E-cadherin下调，N-cadherin、Vimentin上调）的动态变化提示，ARID1B可能通过双重调控（表观遗传重塑与信号通路激活）驱动EMT进程。这与近期发现的SWI/SNF复合物通过抑制ZEB1转录抑制EMT的机制形成有趣对比^[19]^，暗示ARID1B可能存在独立于经典复合物功能的调控网络^[20]^。

值得注意的是，本研究在动物模型中观察到ARID1B缺失显著加速肿瘤生长，并且体外实验显示其促进细胞迁移和侵袭的能力，这种体内外表型的一致性强调了ARID1B在肿瘤微环境调控中的核心地位。研究^[21,22]^提示SWI/SNF复合物可通过调节免疫相关基因[如程序性死亡配体1（programmed death-ligand 1, PD-L1）]影响肿瘤免疫逃逸。本研究中ARID1B缺失是否通过改变细胞因子分泌或免疫细胞浸润影响成瘤能力，仍需进一步探索。

尽管本研究取得了重要发现，仍存在以下局限性。首先，实验仅聚焦于A549和PC9两种腺癌细胞系，未来需在肺鳞癌细胞及原代细胞中验证结果的普适性；目前仅通过WB检测了EMT相关蛋白的表达变化，机制阐述较为肤浅，ARID1B调控NSCLC细胞增殖、迁移和侵袭可能涉及不同分子机制。GPR87被证实可以通过激活RHO/ROCK通路促进NSCLC细胞的侵袭和迁移^[23]^。ARID1B是否也通过类似的通路调控NSCLC细胞的迁移和侵袭，值得进一步探讨。其次，MAPK/PI3K通路激活的具体上游调控机制尚未完全阐明，ARID1B缺失是否通过直接调控通路激酶（如Ras家族）或间接改变染色质开放性影响转录因子结合，需通过ChIP-seq或ATAC-seq等表观基因组学技术深入解析；最后，临床样本中ARID1B表达与临床病理特征、患者预后、MAPK通路激活的相关性仍需大量临床病例验证。

综上所述，本研究揭示了ARID1B基因缺失对NSCLC细胞增殖、迁移和侵袭能力的促进作用，以及ARID1B缺失通过MAPK/EMT轴促进NSCLC进展的机制，不仅拓展了对SWI/SNF复合物功能多样性的认知，也为开发靶向染色质重塑与信号通路交互作用的联合治疗策略提供了理论依据。未来研究可进一步探索ARID1B缺失与其他表观遗传调控因子（如EZH2、HDAC）的协同作用，并在临床样本中验证其作用，为NSCLC的诊断和治疗提供新的策略。
